# 

**Published:** 1880-02-20

**Authors:** 


### TABLE OF CONTENTS.

ORIGINAL AND SELECTED ARTICLES.

Reply to Prof. Joynes' Paper on "Quarantine by the General Government," and to Prof. Chaille on "Castration and Spaying as a Means of Arresting Syphilis," by Dr. Alban S. Payne, Professor of Practice, in Southern Medical College, 41. Some Remarks on Chloroform, by A. Hazlewood, M.D., 46. On Some Points in the Therapy of Chloral Hydrate, by J. W. Hickman, M.D., Delta, Pa., 49. Four Gynecological Facts not Usually Mentioned in Text-Books, by O. E. Herrick, M.D., 52.

ABSTRACTS AND GLEANINGS.

Gun-Shot Wound of the Uterus, 57. External Urethrotomy by Wheelhouse's Method, 58. Permanganate of Potassa in Chronic Otorrhœa, 60. Nitrous Oxide Gas, 61. Belladonna in Obstinate Constipation; Compressed Nitrous Oxide as an Anæsthetic, 62. How to Apply the Hot Water Vaginal Douche; Acute Orchitis, 63. Burn Successfully Treated by Bicarbonate of Soda, 64. Treatment of Strychnia Poisoning; Was the Child Born Premature or at Full Term? 65. The Cold Bath in Infantile Convulsions, 66. Sulphite of Soda in Malarial Fevers; A New Rhinoplastic Operation, 67. How to Cure Fits of Sneezing; Evidences that Dead Infants Were Born Alive, 68. Cause of Ague; Poisonous Vinegar; Morphia Tartrate; New Method in Breech Presentation, 69. Menthol as an Antiseptic; Sclerotic Acid; Inaction of the Bladder; On the Use of Carlsbad Water in Diabetes Mellitus; Carnauba Root; Cayaponin, 70.

SCIENTIFIC ITEMS.

Galvanic Oxidation of Gold; Carrier Pigeons at Great Altitudes, 71. Natural Enemies of the Telegraph; The Colony of Victoria, 72.

PRACTICAL NOTES AND FORMULÆ.

Sulphate of Zinc in Anemia; Dysmenorrhœa, 73. The Purity of Chloroform, 74. Antiodontalgic; Constipation; Alcott's Red Drops, 75. Purgativ[illegible]n Piles; For Acute Rheumatism; A Good Blacking; Remedy for Corns, 76.

EDITORIAL AND MISCELLANEOUS.

Editorial Notices, 77. Book Notices, 79. Receipted; Special Notices, 80.

